# Measure to improve: Is there a patient‐acuity measurement tool suitable for use in maternity service provision in the Netherlands? A systematic review

**DOI:** 10.1002/hsr2.756

**Published:** 2022-10-11

**Authors:** Doug J. Cronie, Ageeth Rosman, Raymond de Vries

**Affiliations:** ^1^ Institute of Health Rotterdam University of Applied Sciences Rotterdam The Netherlands; ^2^ Center for Bioethics and Social Sciences in Medicine University of Michigan Medical School Ann Arbor Michigan USA

**Keywords:** female, measurement‐tool, Midwife, patient‐acuity, staffing

## Abstract

**Background and Aims:**

It is difficult to overestimate the importance of safe staffing levels within the context of effective, quality healthcare. Poor staffing has been cited as a contributory factor in the number of unnecessary hospital deaths. This is particularly so in maternity care, where poor staffing has been inexorably linked to avoidable perinatal and maternal mortality. In the Netherlands, maternity service provision (MSP) is stratified into primary (community)‐ and secondary (hospital)‐based care. While most midwives (71%) work in primary care, the majority are self‐employed or work in small group practices. Where women birth at home, one‐to‐one care during labor is the norm. However, despite the existence of a national standard for birth‐related care, which states unequivocally that women birthing in hospitals should (also) receive one‐to‐one care, while in labor this is not always the case. The extent of compliance with the national care standard has until now not been the subject of scrutiny. We aim to identify evidence for the use of patient‐acuity measurement tools (PAMTs) in MSP to explore the extent of one‐to‐one care for women birthing in hospitals in the Netherlands and select and/or modify a valid PAMT suitable for use in maternity units in hospitals in the Netherlands to assess to what extent the quality standard of one‐to‐one care for birthing women is being met.

**Methods:**

In this systematic literature review, all citations are first screened for title and abstract, then full text for suitability of inclusion.

**Results:**

Three studies were identified for inclusion in the review. One PAMT is recommended.

**Conclusion:**

One PAMT suitable for use in maternity service was identified. However, the evidence level for use is low. Nevertheless, in view of the unique nature of midwifery service provision, a PAMT specifically developed for use in maternity service is preferable.

## 1 INTRODUCTION

It is difficult to overestimate the importance of safe staffing levels within the context of effective, quality healthcare. Poor staffing has been cited as a contributary factor in the number of unnecessary hospital deaths,^1^ an increase in hospital‐acquired infections,^2^ increased length of hospital stays,^3^ and higher levels of staff sickness and turnover.^4^

In the United Kingdom, the importance of adequate staffing in maternity service provision (MSP) has been formally recognized by the establishment of guidance relating to minimum staffing levels.^5^, ^6^, ^7^ The US state of California has gone further, creating a legal mandate to guarantee minimum nurse‐to‐patient ratios.^8^ Safety of care goes hand‐in‐glove with quality. To measure safe staffing levels in MSP, one‐to‐one care for women in labor is a well‐established quality standard,^9^ and in many countries, it is the accepted norm for birthing women.^10^, ^11^, ^12^, ^13^

In the Netherlands MSP is stratified into primary (community)‐ and secondary (hospital)‐based care. Most midwives (71%) work in primary care and the majority are self‐employed or work in small group practices.^14^ A national standard for birth‐related care was published in 2016, which states unequivocally that all birthing women should receive one‐to‐one care while in labor.^15^ Where women birth at home, one‐to‐one care is the norm. However, hospital birth has grown exponentially in the last 25 years and more women now birth in hospitals (73%) than at home.^16^ In addition, women choosing hospital birth in the Netherlands will usually have more complex care needs than those birthing at home.^17^

The hospital care team for birthing women is likely to consist of a mix of maternity care assistants (MCAs), obstetric nurses, clinical (hospital) midwives, and obstetricians. However, no nationally agreed minimum staffing levels for MSP in hospitals in the Netherlands exist and only 29% of midwives are employed in the hospital setting.^14^ The care standard suggests, where appropriate, during the first stage of labor, that [the facilitation of] one‐to‐one care can be delegated to an MCA or obstetric nurse. However, the skill mix of the hospital care team notwithstanding, evidence suggests that women birthing in hospitals in the Netherlands are not always able to access one‐to‐one care while in established labor.^18^, ^19^

The care standard was written in 2016 and published in 2017, with a deadline of 2019 for review and incorporation.^20^ Guidance from the College of Perinatal Care (CPZ) states that by the review date every MSP in the Netherlands should have a policy in place that demonstrates how the core elements of the care standard have been met. The regulations set out in the updated version of the care standard^20^ appear in the advice section of the website of the National Care Institute (NZa) as an incorporated policy.^21^ In addition, the general rules section of the association of insurers (the buyers of pregnancy‐related care in the Netherlands) states unequivocally in their general rules section that “all buyers of pregnancy related care must do so from providers who comply with the regulations as set out by the NZa.”^22^ In any event, all stakeholders subscribe to the notion that (the needs/wants of) birthing women are “central” to service provision. The CPZ goes further in their guidance relating to the evaluation of the implementation of the care standard, stating that this concept—women‐centered care—is the “most important” concept contained within the standard.^20^

To date, however, no audit of staffing numbers has been done. Lacking a national evaluation, it is impossible to know if MSP in the Netherlands is meeting the mandated standard of care. To do such an evaluation, it is necessary to have a measure of patient acuity^23^ related to the number—and skill mix of staff—commonly expressed as “full‐time equivalents” (FTE).

There are a number of validated tools—often referred to as “patient‐acuity measurement tools' (PAMTs)—used for this purpose.^24^ This manner of mathematical modeling is widespread in nursing and has been around for some time.^25^ However, validated PAMTs specifically for use in MSP are less common^26^, ^27^ and, to our knowledge, staffing levels in maternity units in the Netherlands have not been studied. In this paper, we identify and examine the evidence relating to PAMTs designed for use in MSP and assess the suitability of identified PAMTs for use in maternity hospital settings in the Netherlands.

The aims of this research are:

- To identify high‐level evidence, that is, studies that either: (1) compare two methods for measuring patient acuity in maternity‐care settings in an experimental or quasi‐experimental manner, or (2) or systematically reviewed, or performed a meta‐analysis of, experimental studies relating to the use of PAMTs in MSP to explore the extent of one‐to‐one care for women birthing in hospitals in the Netherlands.
- To select a valid patient acuity measurement tool (capable of measuring acuity with a skill mix of differing professionals) suitable for use in maternity units in hospitals in the Netherlands to assess to what extent the quality standard of one‐to‐one care for birthing women is being met.

Null hypothesis: There are enough midwifery care professionals in maternity hospital settings in the Netherlands to be able to facilitate the required standard of one‐to‐one care for all women in established labor.

## 2 METHODS

The scope of this study is limited to (the measurement of) safe staffing levels for birthing women in hospitals in the Netherlands. We used the CPZ care standard of one‐to‐one care for women in labor as a benchmark for the purpose of measuring acuity related to (the availability of) FTE staff. Therefore, in this study, where staffing is mentioned in the context of MSP in the Netherlands, the discussion is limited to midwifery staff and women birthing in hospitals.

**Figure 1:**
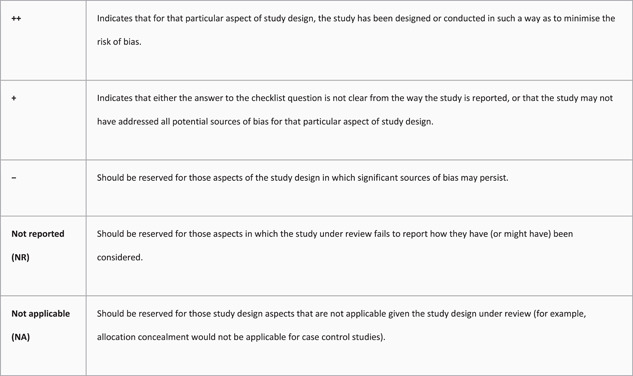
NICE quality appraisal checklist for quantitative intervention studies.^29^ NICE, National Institute for Health and Care Excellence.

As there are no current national guidance documents pertaining to the measurement of safe midwifery staffing levels within the Netherlands, we turned first to the guideline “Safe midwifery staffing for maternity settings” developed for this purpose by the UK National Institute for Clinical Effectiveness.^13^ To locate evidence, we used a systematic search strategy (as described in the National Institute for Health and Care Excellence [NICE] guideline), which was developed for the purpose of identifying midwifery staff requirements and skill mix.^28^ We rated the strength of the evidence located using the NICE Quality appraisal checklist for quantitative intervention studies,^29^ as shown in Figure 1 (also see Supporting Information: Figure 1).

**Figure 2:**
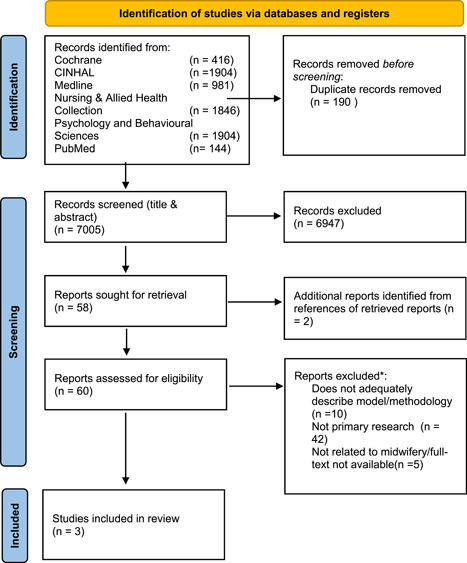
Flowchart search results. *For full list of excluded studies (and reasons for exclusion) see Supporting Information: Appendix 3.

In the absence of a systematic process or existing modeling, frameworks or toolkits for the purpose of measuring staffing levels in MSP in the Netherlands, we started by reviewing the evidence for the use of PAMTs in MSP in other countries. We focused our search on evidence which supports the use of PAMTs specifically designed for use in birth‐care settings and which were used for the assessment of safe staffing levels using the standard of one‐to‐one care for birthing women during labor. Other outcome measures we considered were cost, satisfaction, and care outcomes. The search strategies (and the databases searched) that were used to locate the evidence are listed in Supporting Information: Appendix 1. Since this search was developed and first undertaken by Warttig and Little in 2014^28^ (with permission), we repeated the search to locate any new/additional evidence. We systematically searched six medical databases. The flowchart displayed in Figure 2 lists our findings.

The inclusion criteria were as follows: We began with a search for high‐level evidence, that is, studies that either: (1) compare two methods for measuring patient acuity in maternity‐care settings in an experimental or quasi‐experimental manner, or (2) or systematically reviewed, or performed a meta‐analysis of, experimental studies. In their earlier study, Warttig and Little^28^ limited their search to randomized control trials or studies using an experimental or experimental approach. We did not limit our search to “randomized controlled trials,” to conduct a broader search with the scope of offering additional information.

We excluded studies where the methodology was unclear, where the model of measurement was not adequately described, where the study did not describe a birth‐care setting, where there was insufficient detail to ascertain the manner of measurement or the characteristics of the staff involved, or where the authors failed to describe staffing levels.

The limitations were as follows: As the focus of our study is MSP, in a hospital‐care setting, involving obstetric care professionals, we applied the following filter(s) to our search: “female,” “adult 19–44 years.” In addition, we sought only references where (initially) the abstract was available. Following a review of the title and abstract, if the article was deemed relevant, we reviewed the full text. Since most of the PAMTs require a computer‐based mathematical assessment of acuity related to staffing level, we limited our search to articles <25 years old and to those available in English. Figure 2 shows a flowchart with in/exclusions (see Supporting Information: Figure 2).

Our systematic search of six medical databases resulted in an initial total of 7195 citations. Once duplicates were removed all remaining citations and abstracts were scanned for relevance, 58 citations remained for full‐text review. During the full‐text review, two additional citations were uncovered. Ultimately, three articles met our inclusion criteria (Supporting Information: Appendix 2). Supporting Information: Appendix 3 lists the 57 articles that did not meet our criteria and the reason for their exclusion.

## 3 WHAT THE EVIDENCE TELLS US

**Figure 3:**
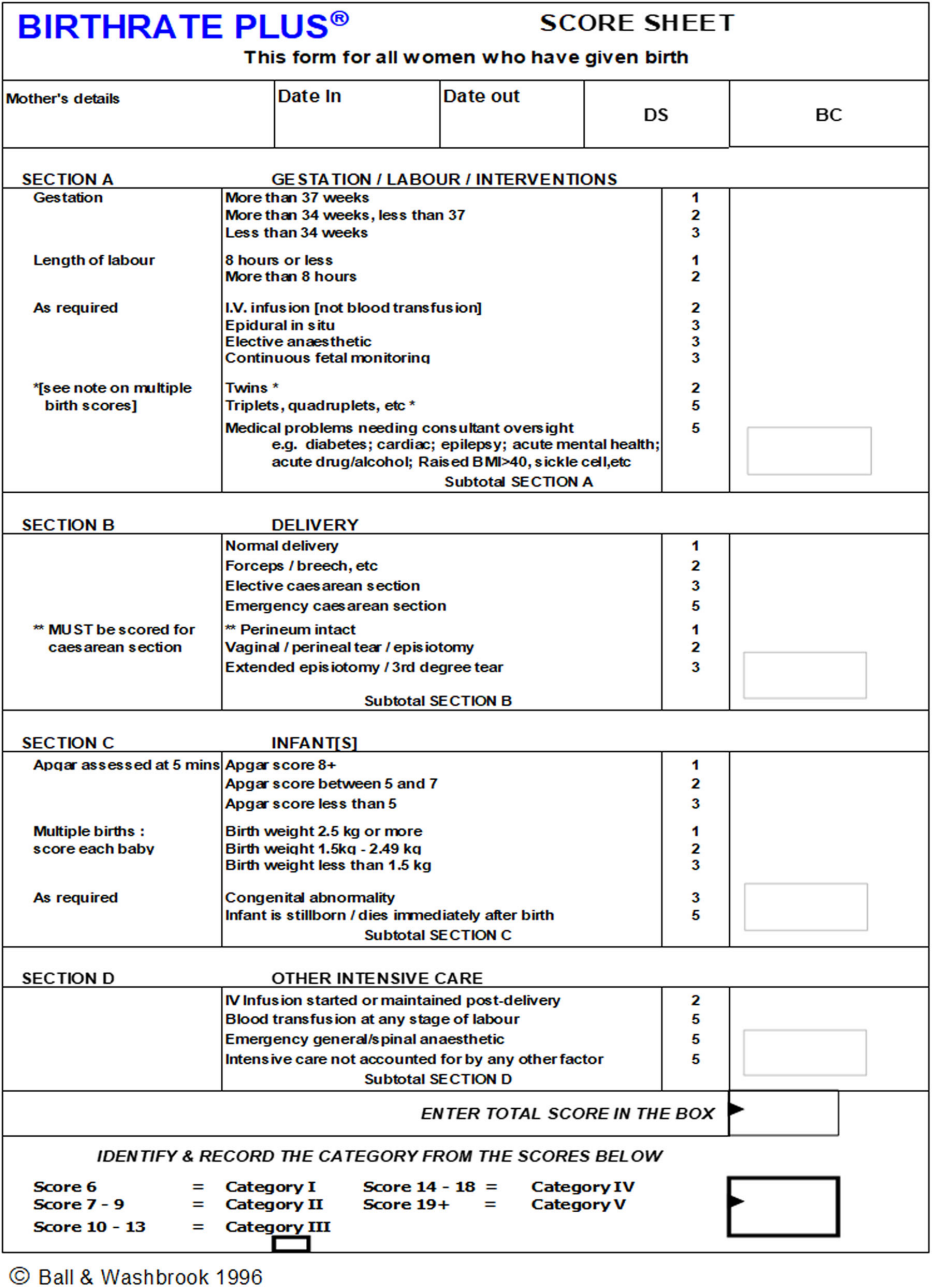
Birthrate+ patient classification scoring tool

Two of the three articles selected for this review^30^, ^31^ used an experimental approach. Both used a computer‐simulated model compared to data obtained using an existing PAMT: Birthrate plus (BR+) to compare the extent to which one‐to‐one care by a midwife for birthing women was provided. BR+ uses a five category scale (1 = *low acuity*, 5 = *high acuity*) to assess the level of acuity of care required by birthing women. BR+ works by using the principle that women with complex health needs will require the care of more than 1 midwife during their labor. While this may vary from case to case, the BR+ methodology applies increased ratios of midwife time as follows: Category 3 has the ratio of 1.2 midwives per woman; Category 4 has a ratio of 1.3; and Category 5 has a ratio of 1.4. Figure 3 gives more detail regarding the BR+ classification tool.

Allios et al.^30^ developed a simulation model using discrete event simulation (DES) based on routinely collected data from a UK hospital maternity service, which had approximately 6000 births per year. DES is an accepted way of measuring resources, processes, and patients, to assess optimal levels of care.^32^ Allios et al.^30^ compared a range of maternity care scenarios. For the purposes of this study, we were primarily concerned with the scenarios modeling one‐to‐one care for birthing women. They found that when using the simulation model approximately 23% of the time there were more women in labor and in operating theater than available midwives, making it impossible to provide one‐to‐one care for all birthing women.

A strength of the study of Allios et al.^30^ is its attention to cost. The modeling indicated that for this hospital three extra midwives per shift (an increase of 30%) would be required to facilitate one‐to‐one care for birthing women 95% of the time. The model also found that staff shortages were more pronounced during the day than at night, suggesting that safe staffing requires careful attention to scheduling.

A limitation of this study is a lack of clarity about how the Trust's actual midwifery staffing requirement had been determined. Using the NICE quality appraisal checklist for quantitative intervention studies, this study has an evidence quality level of (−).

Allen and Thornton^31^ also used an unspecified simulation model, based on data routinely collected from a large UK hospital maternity unit (different to that used by Allios et al.^30^), also with approximately 6000 births per year. They performed a retrospective analysis of labor ward workload based on a comparison of staffing levels using BR+ ata with a computer simulation of different clinical scenarios of labor wards. The variation in births by date and time of day was analyzed over a 1‐year period. Three months of BR+ data were analyzed for variation of workload by case mix. The main outcome was that there were distinct peak periods of work overload (where there were more birthing women than midwives). These occurred more between Monday to Friday and more in daytime hours than in the evening and night. Some of this overload could be explained by the emphasis on planned work (cesarean sections and inductions of labor). While more efficient planning could partially address this problem; nevertheless, the researchers found that the existing staffing calculations of the hospital (1.4 midwives to 1 birthing woman) resulted in work overload 15%–25% of the time. The calculations obtained by modeling suggested an increase in staffing to 1.8:1 would be required to facilitate one‐to‐one care for birthing women 95% of the time. Importantly, the researchers also suggest that this ratio may likely be higher for smaller maternity units (<2000 p.a.).

The interpretation of the results of this study, however, are limited by an inadequate description of the simulation model. Using the NICE quality appraisal checklist for quantitative intervention studies, this study has an evidence quality level of (−).

The remaining article by Zhou et al.^33^ described a large cohort study. The authors used the BR+ PAMT to investigate midwifery services in western China related to the demand for midwives. They analyzed the maternity case records of all women from 28 randomly selected hospitals (*n* = 18,520) who birthed in 2017 and 2018. They found BR+ to be a reliable tool for the measurement of patient acuity and discovered that the adequacy of midwifery service varied between hospitals in western China. The number of birthing women exceeded the calculated availability of midwives in 21% of the hospitals studied. The authors suggest that hospitals with greater concentrations of birthing women requiring higher levels of care under the BR+ classification (see Supporting Information: Figure 3) require a higher midwife‐to‐birthing‐women ratio.

Due to its nonexperimental design, using the NICE quality appraisal checklist for quantitative intervention studies, this study has an evidence quality level of (−).

## 4 DISCUSSION

A binding condition for the provision of MSP in the Netherlands, specified by the organization that regulates health care in that country—the NZa—is that (suppliers of) MSP must comply with the recommendations set out in the national standard for birth‐related care.^20^, ^34^ That standard explicitly states, “women birthing in hospitals should, from the moment of admission, receive one‐to one care from a midwifery care professional.” The care standard suggests that (the provision of) one‐to‐one care can (where appropriate), during the first stage of labor, be delegated to an MCA or obstetric nurse. However, the document goes on to specify the definition of “midwifery care professional” as: a midwife, a physician assistant‐midwife, an obstetrician, an obstetrician‐in‐training, a qualified doctor employed in obstetrics, or a general practitioner schooled in obstetrics.^20^ Currently, there is no measurement of this standard.

The aim of this review was to assess the evidence relating to PAMTs specifically designed for use in MSP and to assess their suitability for use in the Netherlands. The introduction of the use of a PAMT would allow us to audit service in the hospital setting related to the care standard of one‐to‐one care for birthing women.

To inform the process of our review, we used the principles set out by the National Institute of Clinical Effectiveness in their document, Developing NICE guidelines: the manual. Process and methods [PMG20], which were as follows: the review should address an appropriate and clearly focused question, should consider the type of studies relevant to the review, should ensure that the literature search is sufficiently rigorous to identify all the relevant studies, and should assess and report the quality of the studies included.^35^

Based on the evidence we found, PAMTs are in widespread use throughout the spectrum of healthcare. However, in general, the evidence level for their use was low and research into their validity was lacking. BR+, however, is the most widely used PAMT specifically developed for use in MSP. BR+ is thoroughly tested,^36^ widely used,^37^ and recommended by the Royal College of Obstetricians and Gynaecologists, the Royal College of Midwives, the Royal College of Anesthetists, and the Royal College of Paediatrics and Child Health.^38^ In addition, BR+ has more than 20 years of application and is used for the assessment and measurement of safe midwifery staffing by over 100 healthcare Trusts in the United Kingdom as well as being endorsed by the UK National Institute for Clinical Effectiveness.^6^ BR+ can also be used in modeling staffing numbers using a mix of professional disciplines, for example, MCAs and obstetric nurses.^37^ When compared to computer simulation modeling, however, BR+ may underestimate the actual number of FTEs needed to meet the agreed standard.

Notwithstanding their limitations, PAMTs are seen to be an effective way in which to monitor staffing levels and to measure staffing in relation to patient acuity.^39^ However, evidence suggests that the unique nature of midwifery and in particular the need for one‐to‐one care during birth means that the use of PAMTs specifically related to MSP is likely to give a more realistic view of staffing levels than those not specifically developed for this purpose.^37^ BR+ is currently the only MSP‐specific, (inter)nationally used tool that gives the intelligence and insights needed to be able to model midwifery numbers, skill mix and deployment, and to inform decision‐making about safe and sustainable services.^7^

In one study located for this review^31^ where BR+ data were compared to that generated by a computer simulation model, BR+ was found to underestimate the total number of staff needed. This effect was most apparent during peak periods of busyness. In addition, evidence suggests that this underestimation effect may be more pronounced in small (<2000 births per annum) maternity units and in settings where there are more women in maternity units with higher levels of acuity. Nonetheless, the framework of BR+ encompasses all 19 of the recommended steps for a toolkit for the measurement of safe staffing levels,^6^ and in this sense, it is a robust tool and can be recommended.

In any and all events, BR+, when used as part of a systematic process for the measurement of safe staffing, remains the most widely used tool for the measurement of staffing in MSP and can provide a starting point for the audit of the standard of one‐to‐one care for birthing women. In addition, the “Safe midwifery staffing for maternity settings” guideline^6^ recommends that the midwifery staffing establishment for each maternity service should be determined at least every 6 months and that a systematic process should be used to determine the midwifery staffing establishment. This should go some way toward the detection (and any required rectification) when considering variation/underestimation in the calculation of staffing establishments.

In summary, not measuring staffing levels against the published standard: one‐to‐one care for birthing women leaves us unable to audit this standard and unable to demonstrate that service provision best meets women's needs and may thus be derelict.

Given that untoward incidents are on the rise in MSP in the Netherlands^40^ and that elsewhere, when rigorous examination of calamitous incidents has occurred, results often show that unsafe staffing levels have played a fundamental part in each of these instances,^41^ when combined with the findings of this review (i.e., that a robust measurement tool exists), leads us to suggest that the measurement of safe staffing levels in Dutch hospital maternity settings should be incorporated into the existing quality assurance mechanisms and become an ongoing (and permanent) feature of audit of service provision. In doing so, we are mindful that the underlying vision and scope of such a development should be that all women, irrespective of where they choose to birth, deserve high‐quality, one‐to‐one care during labor. Our perspective here is that improving the quality of midwifery care in Dutch maternity hospitals should not be at the cost of quality or service provision norms achieved in primary care.

## 5 STRENGTHS AND WEAKNESSES

We used a systematic approach and search strategy, which was recommended by NICE,^28^ to locate the evidence contained within this review. Further, we used a validated approach^42^ to rate the evidence for this review.

However, the parameters of this review were limited to hospital birthing facilities. In drawing attention to staffing in MSP in hospitals, our findings are not intended to convey suggestions or meaning to staffing in other MSP‐care settings.

On the one hand, BR+ is a thoroughly tested, widely used (and recommended) PAMT, designed specifically for the use of calculating safe staffing levels in MSP settings; on the other hand, BR+ may not accurately predict safe staffing levels 100% of the time; therefore; predictions of safe staffing levels using BR+ may still lead to an underestimate of need. Recommendations relating to any changes in staffing numbers may need to reflect this.

## 6 CONCLUSION

BR+ is the most widely used PAMT for use specifically in midwifery (however, there are gaps in the evidence and overall, the evidence level of located studies is low). Even though BR+ is the most widely PAMT within MSP, it may still result in undermeasurement of safe staffing levels. This effect may be more significant at peak times, such as when more women are having cesarean sections and/or induction of labor. In addition, the undermeasurement effect may be more pronounced in smaller birthing facilities or in clinical settings where the complexity of women's care needs is higher. Therefore, our recommendation of BR+ carries a caveat in that, collectively these factors need to be considered in any and all analyses of safe staffing levels.

In summary, based on our review of the literature, the availability of resources and in the absence of any credible alternative, we feel confident to recommend BR+ in the first instance to benchmark staffing levels in MSP in the Netherlands. We suggest that BR+ is a suitable tool for the assessment of patient acuity in relation to staffing levels in maternity service facilities; to give some indication as to whether the proscribed standard of one‐to‐one care by a midwifery care professional for women birthing in hospitals in the Netherlands is being met.

## AUTHOR CONTRIBUTIONS

**Doug J. Cronie**: Conceptualization; data curation; formal analysis; funding acquisition; investigation; methodology; project administration; writing—original draft; writing—review and editing. **Ageeth Rosman**: Conceptualization; formal analysis; methodology; project administration; supervision; writing—review and editing. **Raymond de Vries**: Conceptualization; methodology; supervision; writing—review and editing.

## CONFLICT OF INTEREST

The authors declare no conflict of interest.

## ETHICS STATEMENT

Ethics approval was sought for this project from the Scientific Committee of OLVG hospital in Amsterdam. Permission was given for the study under reference number: WO21.130.

## TRANSPARENCY STATEMENT

The lead author, Doug Cronie, affirms that this manuscript is an honest, accurate, and transparent account of the study being reported; that no important aspects of the study have been omitted; and that any discrepancies from the study as planned (and, if relevant, registered) have been explained.

## Supporting information

Supporting information.

## Data Availability

The authors confirm that the data supporting the findings of this study are available within the article [and/or] its Supporting information. All data sets generated during and/or analyzed during the current study are available from the corresponding author upon reasonable request.
