# Supplementary material for: Measure to improve: Is there a patient‐acuity measurement tool suitable for use in maternity service provision in the Netherlands? A systematic review
Source: Health Sci Rep. 2022 Oct 11;5(6):e756. doi: 10.1002/hsr2.756 (PMC9552207; doi:10.1002/hsr2.756)
Supplement: Supplementary file 1 — Supporting information. [file HSR2-5-e756-s001.pdf]

Appendix 1. Search strategies (NB. search strategy content was the same for each database. However, specific search terms were revised to suit the search capability of each database).

Search strategy Cochrane database:

ID	Search	Hits
#1	MeSH descriptor: [Hospitals, Maternity] this term only	51
#2	midwife	1438
#3	MeSH descriptor: [Midwifery] this term only	329
#4	MeSH descriptor: [Midwifery] this term only	329
#5	midwi*:ti,ab	2121
#6	MeSH descriptor: [Nurse Midwives] this term only	100
#7	maternity:ti,ab	2059
#8	(intrapartum or postnatal or antenatal or prenatal or perinatal):ti,ab	15872
#9	(birth* or childbirth*):ti,ab	31202
#10	((delivery or labour or labor) near/2 (ward* or suite* or room* or unit*)):ti,ab	1233
#11	MeSH descriptor: [Delivery Rooms] this term only	73
#12	MeSH descriptor: [Birthing Centers] this term only	15
#13	MeSH descriptor: [Perinatal Care] this term only	173
#14	MeSH descriptor: [Prenatal Care] this term only	1529
#15	(msw* not "municipal solid"):ti,ab	241
#16	#1 or #2 or #3 or #4 or #5 or #6 or #7 or #8 or #9 or #10 or #11 or #12 or #13 or #14 or #15	42845

#17 (care near/4 pathway*):ti,ab 893

#18 score card*:ti,ab 14876

#19 scorecard*:ti,ab 29

#20 (acuity near/4 (tool* or score* or system*)):ti,ab 475

#21 bench mark*:ti,ab 160

#22 benchmark*:ti,ab 1306

#23 tool kit*:ti,ab 533

#24 toolkit*:ti,ab 535

#25 dash board*:ti,ab 21

#26 dashboard:ti,ab 234

#27 ((planning or staffing or acuity or severity or need*) near/4 (approach* or model* or system* or tool*)):ti,ab 6150

#28 MeSH descriptor: [Personnel Staffing and Scheduling Information Systems] this term only 1

#29 Safer Nursing Care Tool:ti,ab 18

#30 snct:ti,ab 1

#31 (shelford adj3 tool*):ti,ab 0

#32 aukuh:ti,ab 0

#33 association of UK university hospitals:ti,ab 174

#34 patient care portfolio:ti,ab 70

#35 #17 or #18 or #19 or #20 or #21 or #22 or #23 or #24 or #25 or #26 or #27 or #28 or #29 or #30 or #31 or #32 or #33 or #34 24985

#36 #16 and #35 1027

#37 birthrate plus:ti,ab 0

#38 birth rate plus:ti,ab 2317

#39 (birthrate near/4 tool):ti,ab 0

#40 #37 or #38 or #39 2317

#41 #36 or #40 with Cochrane Library publication date Between Jan 1998 and Jan 2021, 1028

Search strategy Cinhal:

S1

((“Midwifery OR midwi*”.tw. OR “Nurse Midwife”.tw. OR “maternity”.tw.)) OR ((“intrapartum or postnatal or antenatal or prenatal or perinatal”).tw.) OR ((“birth* or childbirth*).tw.) OR (((“delivery or labour or labor”) adj (“ward* or suite* or room* or unit*”)).tw.) OR ((“*Delivery Rooms/ or *birthing centers/))

S2

#s1 AND (care adj3 pathway*).tw. OR ((("score card*").tw. OR (“scorecard*”)).tw.) OR (((acuity adj3 (tool* or score* or system*)).tw.) OR ((("bench mark*").tw. OR (“benchmark*).tw.) OR ((("tool kit*").tw. OR (“toolkit*”)).tw.) OR ((("dash board*").tw. OR (“dashboard”).tw.) OR (((planning or staffing or acuity or severity or need*) adj3 (approach* or model* or system* or tool*)).tw.)

S3

#s1 AND (care adj3 pathway*).tw. OR ((("score card*").tw. OR (“scorecard*”)).tw.) OR (((acuity adj3 (tool* or score* or system*)).tw.) OR ((("bench mark*").tw. OR (“benchmark*).tw.) OR ((("tool kit*").tw. OR (“toolkit*”)).tw.) OR ((("dash board*").tw. OR (“dashboard”).tw.) OR (((planning or staffing or acuity or severity or need*) adj3 (approach* or model* or system* or tool*)).tw.)

Limiters - Published Date: 20000101-20211231; Abstract Available; English Language

Search modes - Boolean/Phrase

Expanders - Apply equivalent subjects Search modes - Boolean/Phrase

Search strategy Medline:

(MH “ Nurse Midwives”) OR (MH “Midwifery+”) OR (MH “perinatal care”) OR (MH “Postnatal care+”) OR (MH “Intrapartum care+”) OR (MH “Prenatal care”) OR (MH “Delivery Rooms”) OR TI midwi* OR AB midwi* OR TI ((intrapartum or postnatal or antenatal or prenatal or perinatal)) OR AB ((intrapartum or postnatal or antenatal or prenatal or perinatal)) OR TI ((birth* or childbirth*)) OR AB ((birth* or childbirth*)) OR TI ((delivery or labour or labor) N1 (ward* or suite* or room* or unit*)) OR AB (((delivery or labour or labor) N1 (ward* or suite* or room* or unit*))) OR TI ((msw* not “municipal solid”)) OR AB ((msw* not “municipal solid”))

AND

TI (care N3 pathway*) or AB (care N3 pathway*) OR TI “score card*” OR AB “score card*” OR TI scorecard* OR AB scorecard* OR TI ((acuity N3 (tool* or score* or system*))) OR AB ((acuity N3 (tool* or score* or system*))) OR TI “bench mark*” OR AB “bench mark*”) OR TI benchmark* or AB benchmark* OR TI “tool kit*” or AB “tool kit*” OR TI toolkit* OR AB toolkit* OR “dash board*” OR AB “dash board*” OR TI dashboard* OR AB dashboard* OR TI (((planning or staffing or acuity or severity or need*) N3 (approach* or model* or system* or tool*))) OR AB (((planning or staffing or acuity or severity or need*) N3 (approach* or model* or system* or tool*))) OR TI “Safer Nursing

care tool” OR AB “Safer Nursing care tool” OR TI snct OR AB snct OR TI aukuh OR AB aukuh OR TI (shelford N3 tool*) OR AB (shelford N3 tool*) OR TI “patient care portfolio” OR AB “patient care portfolio” OR TI “birthrate plus” OR AB “birthrate plus” OR TI (birthrate N3 tool) OR AB (birthrate N3 tool) OR TI “birth rate plus” OR AB “birth rate plus”

Search strategy Nursing & Allied Health Collection:

(midwifery OR midwi*.tw. OR “nurse midwife” OR maternity.tw. OR (intrapartum OR postnatal OR antenatal OR prenatal OR perinatal).tw. OR (birth* or childbirth*).tw. OR ((delivery OR labour OR labor) adj (ward* OR suite* OR room* OR unit*)).tw. OR “*delivery rooms”/ OR “*birthing centres”/ OR exp “*perinatal care”/ OR “*prenatal care”/ OR “maternity support worker”.tw.) AND ((care adj pathway*).tw. OR “score card*”.tw. OR scorecard*.tw. OR (acuity adj (tool* OR score* OR system*)).tw. OR “bench mark*”.tw. OR benchmark*.tw. OR “tool kit*”.tw. OR toolkit*.tw. OR “dash board*”.tw. OR dashboard.tw. OR ((planning OR staffing OR acuity OR severity OR need*) adj (approach* OR model* OR system* OR tool*)).tw. OR “personnel staffing and scheduling information systems”/ OR “safer nursing care tool”.tw. OR snct.tw. OR (shelford adj tool*).tw. OR aukuh.tw. OR “association of UK university hospitals”.tw. OR “patient care portfolio”.tw.) AND (“birthrate plus”.tw. OR “birth rate plus”.tw. OR (birthrate adj tool).tw.)

Search strategy Psychology and Behavioural Sciences Collection:

(midwifery OR midwi*.tw. OR “nurse midwife” OR maternity.tw. OR (intrapartum OR postnatal OR antenatal OR prenatal OR perinatal).tw. OR (birth* or childbirth*).tw. OR ((delivery OR labour OR labor) adj (ward* OR suite* OR room* OR unit*)).tw. OR “*delivery rooms”/ OR “*birthing centres”/ OR exp “*perinatal care”/ OR “*prenatal care”/ OR “maternity support worker”.tw.) AND ((care adj pathway*).tw. OR “score card*”.tw. OR scorecard*.tw. OR (acuity adj (tool* OR score* OR system*)).tw. OR “bench mark*”.tw. OR benchmark*.tw. OR “tool kit*”.tw. OR toolkit*.tw. OR “dash board*”.tw. OR dashboard.tw. OR ((planning OR staffing OR acuity OR severity OR need*) adj (approach* OR model* OR system* OR tool*)).tw. OR “personnel staffing and scheduling

information systems"/ OR “safer nursing care tool”.tw. OR snct.tw. OR (shelford adj tool*).tw. OR aukuh.tw. OR “association of UK university hospitals”.tw. OR “patient care portfolio”.tw.) AND (“birthrate plus”.tw. OR “birth rate plus”.tw. OR (birthrate adj tool).tw.)

Search strategy PubMed:

"((""Midwifery""[Mesh] OR ""Midwifery""[tiab] OR Midwives[tiab] OR Midwife[tiab] OR ""Advanced Practice Nursing""[Mesh] OR ""Advanced Practice Nursing""[tiab] OR ""Nurse Midwives""[Mesh] OR ""Nurse Midwives""[tiab] OR Nurse-Midwive*[tiab] OR Nurse-Midwife[tiab] OR Nurse Midwife[tiab]) AND (((""Personnel Staffing and Scheduling Information Systems""[Mesh] OR ""Personnel Staffing and Scheduling Information Systems""[tiab] OR ""Personnel Staffing Scheduling Information Systems""[tiab] OR ""Personnel Staffing Information Systems""[tiab]) OR (""Continuity of Patient Care""[Mesh] OR ""Continuity of Patient Care""[tiab] OR ""Patient Care Continuity""[tiab] OR ""Continuum of Care""[tiab] OR ""Care Continuum""[tiab] OR ""Continuity of Care""[tiab] OR ""Care Continuity""[tiab]))",,"Abstract, Clinical Trial, Meta-Analysis, Randomized Controlled Trial, Review, Systematic Review, Female, from 1998 - 2021","((""Midwifery""[MeSH Terms] OR ""Midwifery""[Title/Abstract] OR ""Midwives""[Title/Abstract] OR ""Midwife""[Title/Abstract] OR ""Advanced Practice Nursing""[MeSH Terms] OR ""Advanced Practice Nursing""[Title/Abstract] OR ""Nurse Midwives""[MeSH Terms] OR ""Nurse Midwives""[Title/Abstract] OR ""nurse midwive*""[Title/Abstract] OR ""Nurse-Midwife""[Title/Abstract] OR ""Nurse-Midwife""[Title/Abstract]) AND (""Personnel Staffing and Scheduling Information Systems""[MeSH Terms] OR (""Continuity of Patient Care""[MeSH Terms] OR ""Continuity of Patient Care""[Title/Abstract] OR ""Patient Care Continuity""[Title/Abstract] OR ""Continuum of Care""[Title/Abstract] OR ""Care Continuum""[Title/Abstract] OR ""Continuity of Care""[Title/Abstract] OR ""Care Continuity""[Title/Abstract]))) AND ((fha[Filter]) AND (clinicaltrial[Filter] OR meta-analysis[Filter] OR randomizedcontrolledtrial[Filter] OR review[Filter] OR systematicreview[Filter]) AND (female[Filter]) AND (1998:2021[pdat]))",128,07:01:39

Appendix 2. Included Studies:

Allen, M., & Thornton, S. (2013). Providing one-to-one care in labour. Analysis of 'Birthrate Plus' labour ward staffing in real and simulated labour ward environments. BJOG: An International Journal of Obstetrics & Gynaecology, 120(1), 100-107. INCLUDED: Trial comparing two methods of measurement of patient acuity in a maternity setting

Allios M, Cozzi E, McBride T, Palmer W (2014). Modelling of maternity services in England. London. National Audit Office. INCLUDED: Trial comparing two methods of measurement of patient acuity in a maternity setting

Zhou, N., Lu, H., Zhao, H., Li, F., & Yang, M. (2019). Midwifery service and midwifery human resource demand in western China: a cross-sectional study. [Article]. Lancet, 394, S34-S34. INCLUDED: Not an experimental design however, sufficient detail given to be able to assess model

Appendix 3. Excluded studies:

Anca, R., Ruff, D., & Allegra, J. (2019). Development of a Patient Assignment Acuity Tool to Improve Equity in Assignments, Patient Safety, and Nurse Satisfaction in a Labor and Delivery Unit...2019 AWHONN Convention, June 8-12, 2019, Atlanta, Georgia. *JOGNN: Journal of Obstetric, Gynecologic & Neonatal Nursing, 48*, S19-S19. EXCLUDED: poster presentation. Does not describe the model of measurement

Andersson, P.-Å., Bruce, D., Walander, A., & Viberg, I. (2011). Time for a new budget allocation model for hospital care in Stockholm? *Health care management science, 14*(1), 36-55. EXCLUDED: the model of measurement was not adequately described

Ashcroft, B., Elstein, M., Boreham, N., & Holm, S. (2003). Prospective semistructured observational study to identify risk attributable to staff deployment, training, and updating opportunities for midwives. *BMJ (Clinical research ed.), 327*(7415), 584. EXCLUDED: the methodology was unclear

Baldwin, J., Brodrick, A., Cowley, S., & Mason, N. (2009). Implementing. Maternity matters: using the toolkit as a pathway to success. *Midwives, 12*(1), 36-37. EXCLUDED: not primary research, description only

Ball, J., Bennett, B., Washbrook, M., & Webster, F. (2003). Birthrate Plus Programme. Factors affecting staffing ratios. *British Journal of Midwifery, 11*(6), 357-360. EXCLUDED: not primary research, description only

Bingham, D., & Ruhl, C. (2015). Planning and evaluating evidence-based perinatal nurse staffing. *Journal of obstetric, gynecologic, and neonatal nursing : JOGNN, 44*(2), 290-308. EXCLUDED: not primary research, description only

Birthrate Plus delivers for mothers and midwives. (2014). *Lamp, 71*(2), 12-13. EXCLUDED: not primary research, description only

Birthrate Plus takes into account case mix variations. (2011). *Lamp, 68*(3), 14-14. EXCLUDED: not primary research, description only

Bishop, T. (2019). Short sighted approach to workforce planning? *Practice Nurse, 49*(12), 5-5. EXCLUDED: not primary research, description only

Buchan, J. (2009). Achieving workforce growth in UK nursing: policy options and implications. *Collegian (Royal College of Nursing, Australia), 16*(1), 3-9. EXCLUDED: not primary research, description only

Christopher R Burton, Jo Rycroft-Malone, Lynne Williams, Siân Davies, Anne McBride, Beth Hall, Anne-Marie Rowlands, Adrian Jones, Denise Fisher, Margaret Jones and Maria Caulfield. NHS managers' use of nursing workforce planning and deployment technologies: a realist synthesis HEALTH SERVICES AND DELIVERY RESEARCH VOLUME 6 ISSUE 36 NOVEMBER 2018 ISSN 2050-4349 EXCLUDED: not primary research, description only

Call for new approach to staffing in maternity units. (2011). *Practising Midwife, 14*(4), 6-6. EXCLUDED: not primary research, description only

Camur, D., Oztek, Z., & Caman, O. K. (2010). An estimation method for midwife demand. *Cahiers de sociologie et de demographie medicales, 50*(4), 529-539. EXCLUDED: insufficient detail given regarding model used

Carlough, M., Chetwynd, E., Muthler, S., & Page, C. (2021). Maternity Units in Rural Hospitals in North Carolina: Successful Models for Staffing and Structure. *Southern Medical Journal, 114*(2), 92-97. EXCLUDED: not primary research, description only

Castro Lopes, S., Titulaer, P., Bokosi, M., Homer, C. S. E., & ten Hoope-Bender, P. (2015). The involvement of midwives' associations in policy and planning about the midwifery workforce: A global survey. *Midwifery, 31*(11), 1096-1103. EXCLUDED: not primary research, description only

Connor, J., LaGrasta, C., & Hickey, P. (2018). Scaling Up and Validating a Nursing Acuity Tool to Ensure Synergy in Pediatric Critical Care...2018 National Teaching Institute Research Abstracts Presented at the AACN National Teaching Institute in Boston, Massachusetts, May 21-24, 2018. *American Journal of Critical Care, 27*(3), e2-e3. EXCLUDED: conference abstract

Draper, E. S., Manktelow, B. N., McCabe, C., & Field, D. J. (2004). The potential impact on costs and staffing of introducing clinical networks and British Association of Perinatal Medicine standards to the delivery of neonatal care. *Archives of Disease in Childhood -- Fetal & Neonatal Edition, 89*(3), F236-240. EXCLUDED: not primary research, description only

Einerson, B. D. (2018). Coverage and capacity: addressing the 'night & weekend effect' in obstetrics. *BJOG: An International Journal of Obstetrics & Gynaecology, 125*(7), 892-892. EXCLUDED: does not refer to midwives

Flynn, B., Kellagher, M., & Simpson, J. (2010a). Workload and workforce planning: tools, education and training. [Journal Article]. *Nursing Management - UK, 16*(10), 32-35. EXCLUDED: not primary research, description only

Flynn, B., Kellagher, M., & Simpson, J. (2010b). Workload and workforce planning: tools, education and training...second of five articles. *Nursing Management - UK, 16*(10), 32-35. EXCLUDED: not primary research, description only

Hauck, Y. L., Bayes, S. J., & Robertson, J. M. (2012). Addressing the workplace needs of Western Australian midwives: a Delphi study. *Australian Health Review, 36*(2), 9-9. EXCLUDED: does not use a model or toolkit

Hunter, B., Fenwick, J., Sidebotham, M., & Henley, J. (2019). Midwives in the United Kingdom: Levels of burnout, depression, anxiety and stress and associated predictors. *Midwifery, 79*, N.PAG-N.PAG. EXCLUDED: does not consider toolkit or model

Hurley, J., & Dickson, K. (1998). Clinical. Assessing midwifery workload on a labour ward. *British Journal of Midwifery, 6*(7), 444-449. EXCLUDED: Full text could not be located

Isken, M. W., Ward, T. J., Littig, S. J., Isken, M. W., Ward, T. J., & Littig, S. J. (2011). An open-source software project for obstetrical procedure scheduling and occupancy analysis. *Health Care Management Science, 14*(1), 56-73. EXCLUDED: not primary research, description only

Ivory, C. H. (2015). The role of health care technology in support of perinatal nurse staffing. *Journal of obstetric, gynecologic, and neonatal nursing : JOGNN, 44*(2), 309-316. EXCLUDED: not primary research, description only

Jenkin-Cappiello, E. (2000). Oh baby!...a labor and delivery staffing system measures patient census and acuity. *Nursing Management, 31*(2), 35-37. EXCLUDED: not primary research, description only

Jones, M. L. H., Filip, S. J., Smith, H., & Remsburg-Bell, E. (2002). Strategy management system in perinatal services: the role of a patient resource manager. *Lippincott's case management : managing the process of patient care, 7*(1), 27-42. EXCLUDED: does not describe midwifery setting

Kellagher, M., Simpson, J., Flynn, B., & Armstrong, D. (2010). Workload and workforce planning: developing a learning toolkit. [Article]. *Nursing Management - UK, 17*(1), 32-34. EXCLUDED: not primary research, description only

Kildea, S., Larsson, M., & Govind, S. (2012). A review of midwifery in Mongolia utilising the ‘Strengthening Midwifery Toolkit’. *Women & Birth, 25*(4), 166-173. EXCLUDED: does not describe model

Kozhimannil, K. B., Casey, M. M., Hung, P., Han, X., Prasad, S., & Moscovice, I. S. (2015). The Rural Obstetric Workforce in US Hospitals: Challenges and Opportunities. *The Journal of rural health : official journal of the American Rural Health Association and the National Rural Health Care Association, 31*(4), 365-372. EXCLUDED: not primary research, description only

Lankford, D. N. (2013). The Art of Staffing in Labor and Delivery: A Tool to Quantify Staffing Demands. *JOGNN: Journal of Obstetric, Gynecologic & Neonatal Nursing, 42*, S64-S64. EXCLUDED: Not primary research does not describe model

Lockhart, K., Barkby, I., & Kellagher, M. (2010a). Workload and workforce planning: taking a national approach. [Journal Article]. *Nursing Management - UK, 16*(9), 32-34. EXCLUDED: not primary research, description only

Lockhart, K., Barkby, I., & Kellagher, M. (2010b). Workload and workforce planning: taking a national approach...first of five articles. *Nursing Management - UK, 16*(9), 32-34. EXCLUDED: not primary research, description only

Lopes, S. C., Titulaer, P., Bokosi, M., Homer, C. S. E., & ten Hoope-Bender, P. (2015). The involvement of midwives' associations in policy and planning about the midwifery workforce: A global survey. *Midwifery, 31*(11), 1096-1103. EXCLUDED: not primary research, description only

Lowe, C (2015) Validation of an Acuity Measurement Tool for Maternity Services. World Academy of Science, Engineering and Technology International Journal of Medical and Health Sciences Vol:9, No:5, 2015. EXCLUDED: Unclear if model was developed for midwifery and inadequate description of how comparison data was generated

McIlhone, B. (2010). MERAS Report (pp. 18-18): New Zealand College of Midwives. EXCLUDED: not primary research, description only

McKenna, H., & Hasson, F. (2002). A study of skill mix issues in midwifery: a multimethod approach. *Journal of advanced nursing, 37*(1), 52-61. EXCLUDED: not primary research, description only

Morin, H. E. (2017). Development of a Staffing Activity Tool That Works. *JOGNN: Journal of Obstetric, Gynecologic & Neonatal Nursing, 46*, S8-S8. EXCLUDED: not primary research, description only

NSW NURSES AND MIDWIVES RAMP UP ACTION ON UNSAFE STAFFING. (2018). [Article]. *Australian Nursing & Midwifery Journal, 26*(3), 12-12. EXCLUDED: not primary research, description only

O'Brien-Pallas, L., Birch, S., & Murphy, G. T. (2001). Workforce planning and workplace management. *International Nursing Perspectives, 1*(2-3), 55-65. EXCLUDED: not primary research, description only

O'Sullivan, S. (1999). Working to plan: workforce planning in midwifery...Birthrate Plus... workforce planning tool. *RCM Midwives Journal, 2*(7), 216-217. EXCLUDED: not primary research, description only

Research activity by the INO for the Commission on the Implementation of a 35 Hour Week for Nurses & Midwives. (2008). *World of Irish Nursing & Midwifery, 16*(11), [20]-[20]. EXCLUDED: not primary research, description only

Rice Simpson, K. (2013). Perinatal Acuity Tool. *MCN: The American Journal of Maternal Child Nursing, 38*(3), 191-191. EXCLUDED: not primary research, description only

Sentilhes, L., Galley-Raulin, F., Boithias, C., Sfez, M., Goffinet, F., Le Roux, S., et al. (2020). Staffing needs for unscheduled activity in obstetrics and gynecology. *European journal of obstetrics, gynecology, and reproductive biology, 245*, 19-25. EXCLUDED: does not describe a model or toolkit

Simpson, K. R. (2015). Predicting Nurse Staffing Needs for a Labor and Birth Unit in a Large-Volume Perinatal Service. *JOGNN: Journal of Obstetric, Gynecologic & Neonatal Nursing, 44*(2), 329-338. EXCLUDED: not primary research, description only

Tawfik, D. S., Profit, J., Lake, E. T., Liu, J. B., Sanders, L. M., & Phibbs, C. S. (2020). Development and use of an adjusted nurse staffing metric in the neonatal intensive care unit. *Health Services Research, 55*(2), 190-200. EXCLUDED: Although the model description was comprehensive, does not describe an experimental study and does not describe a midwifery setting

The BPF works -- when it is used appropriately. (2011). *Queensland Nurse, 30*(2), 24-24. EXCLUDED: not primary research, description only

Thompson, H., Legorreta, K., Maher, M. A., & Lavin, M. M. (2016). PLANNING, DESIGNING, BUILDING, AND MOVING A LARGE VOLUME MATERNITY SERVICE TO A NEW LABOR AND BIRTH UNIT. *MCN: The American Journal of Maternal Child Nursing, 41*(6), 322-331. EXCLUDED: not primary research, description only

Torbet, J. L., Mulkeen, M., Stringer, L., & Fitzpatrick, K. (2015). Development of a Nurse Staffing Model to Accurately Reflect Complexity and Intensity of Patient Care Needs in an Urban Academic Medical Center...Proceedings of the 2015 AWHONN Convention. *JOGNN: Journal of Obstetric, Gynecologic & Neonatal Nursing, 44*, S40-S40. EXCLUDED: Poster presentation, does not describe primary research

Troubleshooters for Birthrate Plus. (2001). *Nursing Standard, 15*(28), 8-8. EXCLUDED: not primary research, description only

Tucker, J., Hundley, V., Kiger, A., Bryers, H., Caldow, J., Farmer, J., et al. (2005). Sustainable maternity services in remote and rural Scotland? A qualitative survey of staff views on required skills, competencies and training. *Quality & safety in health care, 14*(1), 34-40. EXCLUDED: not primary research, description only

Walsh, D. (2006). The challenge of interdisciplinary collaboration: midwifery needs a model. *British Journal of Midwifery, 14*(9), 549-549. EXCLUDED: does not describe a model or toolkit

'We're very keen to see Birthrate Plus in place'. (2011). *Lamp, 68*(3), 14-14. EXCLUDED: not primary research, description only

What's in the nurse staffing ratio claim: Summary of the 2010 Claim for Nurse Staffing Ratios and Skill Mix for Safe Patient Care. (2010). *Lamp, 67*(8), 20-21. EXCLUDED: not primary research, description only

Whitehead, L., & Myers, H. (2016). The effect of hospital nurse staffing models on patient and staff-related outcomes. *International Journal of Nursing Practice (John Wiley & Sons, Inc.), 22*(4), 330-332. EXCLUDED: does not describe a maternity setting

Wilson, B. L., & Butler, R. J. (2021). Identifying optimal labor and delivery nurse staffing: The case of cesarean births and nursing hours. *Nursing outlook, 69*(1), 84-95. EXCLUDED: not primary research, description only

Wilson B, Blegan M. Labor and Delivery Nurse Staffing as a Cost-Effective Safety Intervention, October 2010The Journal of perinatal & neonatal nursing 24(4):312-9, DOI: 10.1097/JPN.0b013e3181f32703 EXCLUDED: not primary research, description only
